# Electrical stimulation of the auricular branch of the vagus nerve potentiates analgesia induced by physical exercise in mice with peripheral inflammation

**DOI:** 10.3389/fnint.2023.1242278

**Published:** 2023-10-12

**Authors:** Aline Raulino Dutra, Daiana Cristina Salm, Rafaela Hardt da Silva, Fernanda Tanaka, Daniela Dero Lutdke, Bruna Hoffmann de Oliveira, Rose Lampert, Edsel B. Bittencourt, Gianluca Bianco, Vinícius M. Gadotti, William R. Reed, Josiel Mileno Mack, Franciane Bobinski, Ari O. O. Moré, Daniel Fernandes Martins

**Affiliations:** ^1^Experimental Neuroscience Laboratory (LaNEx), Postgraduate Program in Health Sciences, University of Southern Santa Catarina, Palhoça, SC, Brazil; ^2^Coastal Health Institute, Jacksonville, FL, United States; ^3^Research Laboratory of Posturology and Neuromodulation RELPON, Department of Human Neuroscience, Sapienza University and Istituto Di Formazione in Agopuntura E Neuromodulazione IFAN, Rome, Italy; ^4^Department of Clinical Neurosciences, Hotchkiss Brain Institute, Alberta Children’s Hospital Research Institute, University of Calgary, Calgary, AB, Canada; ^5^Department of Physical Therapy, University of Alabama at Birmingham, Birmingham, AL, United States; ^6^Rehabilitation Science Program, University of Alabama at Birmingham, Birmingham, AL, United States; ^7^Integrative Medicine and Acupuncture Division, University Hospital, Federal University of Santa Catarina, Florianópolis, Santa Catarina, Brazil

**Keywords:** autonomic system, exercise, inflammatory pain, parasympathetic, integrative medicine

## Abstract

**Objective:**

This study evaluated the antihyperalgesic and anti-inflammatory effects of percutaneous vagus nerve electrical stimulation (pVNS) associated with physical exercise, i.e., swimming, in mice with peripheral inflammation.

**Methods:**

The pain model was induced by intraplantar (i.pl.) injection of Freund’s complete adjuvant (CFA). Sixty-four male Swiss mice (35–40 g) received an i.pl. of CFA and underwent behavioral tests, i.e., mechanical hyperalgesia, edema, and paw temperature tests. Additionally, cytokine levels, specifically interleukin-6 (IL-6) and interleukin-10 (IL-10), were determined by enzyme-linked immunosorbent assay. Mice were treated with swimming exercise for 30 min alone or associated with different time protocols (10, 20, or 30 min) of stimulation in the left ear with random frequency during four consecutive days.

**Results:**

pVNS for 20 min prolonged the antihyperalgesic effect for up to 2 h, 24 h after CFA injection. pVNS for 30 min prolonged the antihyperalgesic effect for up to 7 h, 96 h after CFA injection. However, it did not alter the edema or temperature at both analyzed times (24 and 96 h). Furthermore, the combination of pVNS plus swimming exercise, but not swimming exercise alone, reduced IL-6 levels in the paw and spinal cord, as well as IL-10 levels in the spinal cord.

**Conclusion:**

pVNS potentiates the analgesic effect induced by swimming, which may be, at least in part, mediated by the modulation of inflammatory cytokines in the periphery (paw) and central nervous system (spinal cord). Therefore, the combination of these therapies may serve as an important adjunctive treatment for persistent inflammatory pain.

## 1. Introduction

Inflammation, when unresolved, contributes to the development of a series of inflammatory diseases (Caravaca et al., 2022). Among diseases of an inflammatory nature, there is osteoarthritis, which is defined as a joint pathology characterized by chronic degeneration of the joint cartilage associated with inflammation of the synovial membrane with formation of osteophytes and sclerosis of the subchondral bone (Shen et al., 2017), being considered the eleventh cause of physical disability in the world (Palazzo et al., 2016; Vina and Kwoh, 2018). The main symptoms triggered by osteoarthritis are pain, joint stiffness and physical disability/loss of function that led to reduced quality of life, anxiety disorders and depression (Scopaz et al., 2009).

Physical exercise is among the non-pharmacological approaches described for the treatment of osteoarthritis capable of promoting analgesia and resolution of inflammation (Goh et al., 2019; Zeng et al., 2021). Physical exercise, in addition to contributing to the reduction of pain, also contributes to the improvement of physical function and quality of life when practiced regularly (Fransen et al., 2015). Still, it is also capable of contributing to the improvement of cardiorespiratory function, increasing muscle strength, stabilizing posture and improving mental health (Garber et al., 2011).

The vagus nerve is the tenth pair of cranial nerves and regulates the body’s homeostasis by acting on the cardiovascular, respiratory, gastrointestinal, endocrine and immune systems (Berthoud and Neuhuber, 2000). By acting on the immune system, the vagus nerve modulates the inflammatory process through three pathways: (i) hypothalamic-pituitary-adrenal axis (Tracey, 2009; Howland, 2014); (ii) splenic sympathetic anti-inflammatory drug (Tracey, 2007; Olofsson et al., 2012); and (iii) cholinergic anti-inflammatory (Tracey, 2007, 2009).

It is well established that physical exercise (Gourine and Ackland, 2019) and non-invasive percutaneous electrical stimulation of the auricular branch of the vagus nerve (pVNS) are effective in increasing vagal activity (Bonaz et al., 2016). Interestingly, these two therapies also contribute to pain relief (Vina and Kwoh, 2018), inflammation reduction (Nesbitt et al., 2015; Frangos and Komisaruk, 2017), and inflammation resolution (Rosas-Ballina and Tracey, 2009). Our research group has previously shown that both the physical exercise of swimming and pVNS reduce the mechanical hyperalgesia caused by CFA alone (Salm et al., 2023). However, the effect of combining the two vagal activation therapies, as has been done in clinical practice, has yet to be investigated.

Given the similarity of the vagus nerve-mediated mechanism of action of the effects of physical exercise and VNS, the purpose of this study was to use behavioral and biochemical tests to evaluate the antihyperalgesic and anti-inflammatory effect of physical exercise associated with pVNS in a mouse model of inflammation.

## 2. Materials and methods

### 2.1. Animals

The present study is characterized as a non-clinical experimental study. Sixty-four male Swiss mice (35–40 g), obtained from the Central Animal House of the Federal University of Santa Catarina (UFSC, Florianópolis, Santa Catarina, Brazil) were used. All animals were housed in collective cages at a temperature of approximately 22°C, with a 12-h light/12-h dark cycle (light from 7 a.m.), with free access to food and water. Before the evaluations, the animals were acclimated in the experimentation room for at least 1 h before the tests, always in the morning. This research was approved by the Committee on Ethics in the Use of Animals (CEUA) of the University of Southern Santa Catarina (CEUA-UNISUL) under protocol 20.007.4.08.IV. All animal care and experimental procedures were performed in accordance with the laboratory animal care guide and by the Brazilian College of Animal Experimentation (Zimmermann, 1983).

The evaluators were blinded to conduct the experiments. The exclusion and euthanasia criteria were as follows: signs of inability to ingest water/feed, immobility, epidermal damage, seizures or vocalization without stimulation. On the day of the experiment, the animals were randomly distributed between groups using the randomization.com automatic generator. Protocols, such as adequate containment in some stages, were also adopted to minimize the effects generated by handling the animals.

### 2.2. Animal model of persistent inflammatory hyperalgesia

To induce the peripheral inflammation model, the animals received an intraplantar injection of CFA (Complete Freund Adjuvant; Sigma-Aldrich, St. Louis, MO, USA) in a 50% solution, dissolved in saline solution and Tween^®^80. Each animal received 20 μl of this solution in the right hind paw 1 day before starting the treatment (Salm et al., 2023).

### 2.3. Swimming and percutaneous vagus nerve electrical stimulation (pVNS) treatments

For swimming, the animals were placed in a plastic box measuring 540 mm × 390 mm × 325 mm, divided with acrylic into eight compartments of 170 mm × 110 mm each, containing approximately 35 L of water at 37°C. 1 ml of neutral liquid shampoo was added to each compartment in order to reduce the surface tension and avoid the floating behavior of the animals. Each swimming session lasted 30 min and after finishing the session, the animals were placed in clean shavings to facilitate drying of the body (Mazzardo-Martins et al., 2010).

The animals were habituated to swimming 4 days before the intraplantar injection of CFA. On the first day, they were placed in the water for 30 s twice a day, on the second day for 2 min, also twice a day and on the third and fourth days for 5 min once a day (Mazzardo-Martins et al., 2010).

Groups with pVNS received stimulation alone or in combination with swimming after 30 min of exercise. Treatment with pVNS was performed using the NKL-608 electrostimulation device (NKL electronic products LTDA, Brusque, SC, Brazil). The animals were lightly sedated with 2% inhaled isoflurane and 100% oxygen through a nasal mask. As soon as they were sedated, needling (0.18 mm × 8 mm) was performed on the left ear in region A2 and B2 of the concha cavity 15. Each needle was connected to a cable of the electrostimulation device. We used the random frequency (random noise) in an interval between F1 and F2. The programmed stimuli varied at random frequencies within an interval established between 2 and 10 Hz. Advanced type stimulation was selected; “Noise” type, F1 frequency = 2 Hz, F2 frequency = 10 Hz, unbiased pulse shape, pulse width 200 μs. In random frequency programming, the pulse morphology is of the alternating biphasic type. The treatment times were 10, 20, and 30 min (min) and the intensity of 0.8 mA. The treatment was carried out for four consecutive days (Salm et al., 2023).

After habituation, the animals were randomized to the following groups: CFA (control), swimming, pVNS 10 min, pVNS 20 min, pVNS 30 min, swimming + pVNS 10 min, swimming + pVNS 20 min and swimming + pVNS 30 min. Each group with 8 animals totaling 64 animals. The swimming treatment was performed for 4 consecutive days of swimming session for 30 min.

### 2.4. Study design

**FIGURE 1:**
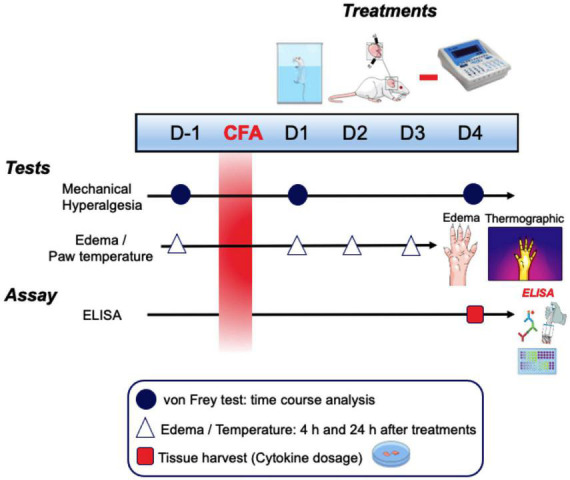
Schematic representation of the experimental design. CFA, Freund’s complete adjuvant; D, days; h, hours.

At 24 and 96 h after CFA injection in the animals’ right hind paw, different treatment times (10, 20, and 30 min) of electrical stimulation on the auricular branch of the vagus nerve in the left ear associated with swimming (30 min) was performed to verify the effect on mechanical hyperalgesia, evaluated by the von Frey test (vF). The effect of pVNS associated with swimming on paw edema and surface temperature was evaluated at 4 and 24 h after treatment daily until the 3rd day. Finally, concentrations of pro- and anti-inflammatory cytokines in the skin of the right hind paw and in the spinal cord were measured at 96 h after CFA injection (Figure 1).

Groups used for the experiments were as follows: CFA + pVNS*off* (CFA injection in the right hind paw and without electrostimulation, only needling for 30 min), Swimming (CFA injection in the right hind paw and swimming exercise for 30 min), pVNS 10 min (CFA injection in the right hind paw and with electrostimulation for 10 min), pVNS 20 min (CFA injection in the right hind paw and with electrostimulation for 20 min), pVNS 30 min (CFA injection in the right hind paw and with electrostimulation for 30 min), Swimming + pVNS 10 min (CFA injection in the right hind paw and Swimming for 30 min with electrostimulation for 10 min), Swimming + pVNS 20 min (CFA injection in the right hind paw and Swimming for 30 min with electrostimulation for 20 min), Swimming + pVNS 30 min (CFA injection in the right hind paw and Swimming for 30 min with electrostimulation for 30 min).

### 2.5. Sample size

The calculation presents the following equation to obtain a confidence coefficient of 95%: *n* = {[(z alpha + z beta) × s]/sigma}. Thus, the number of animals used was the minimum necessary to demonstrate the effects obtained by the treatment (*n*/total = 64), requiring the number of 8 animals per group, based on Daniel (2008).

### 2.6. Assessment of mechanical hyperalgesia

The evaluation of mechanical hyperalgesia was performed using a von Frey monofilament (0.6 g) and only animals that presented a response percentage of around 20% were selected. The animals were evaluated individually on an acrylic chamber (9 cm × 7 cm × 11 cm) without a bottom and covered with a lid on a platform (70 cm × 40 cm) made of wire mesh with a 6 mm mesh so that the monofilament was applied to the plantar surface of the right hind paw. In the 3 days that preceded the injection of CFA, the animals were habituated to an acrylic chamber and wire mesh platform and were stimulated with a filament of 0.2 g, once a day. The animals were submitted to this evaluation and on the following day, and after receiving the intraplantar injection of CFA at 0.5, 1, 2, 3 h and until the effect of the treatment persists (Bobinski et al., 2011).

### 2.7. Assessment of edema and paw surface temperature

Edema and paw surface temperature were measured as the difference from baseline and subsequent measurements of the right hind paw. An Insight brand micrometer was used to measure edema and a Thermal Imager (Testo 880^®^) thermographic camera was used to measure paw surface temperature. Measurements were performed pre and post CFA intraplantar injection and before and 4 h post-treatments, during four consecutive days of treatment (Mazzardo-Martins et al., 2018).

### 2.8. Enzyme-linked immunosorbent assay

To analyze of IL-6 and IL-10 concentrations, 30 min after treatment (of each animal), animals were euthanized, and tissue samples were collected by dissection from the following: skin of the right hind paw and lumbar spinal cord (whole segment L3–L5 at 96 h after CFA injection). Samples were placed inside 2-mL Eppendorf microtubes and stored at a −80°C freezer for posterior analysis. For determination of cytokines concentrations, 100 μL of each sample were used. These cytokine concentrations analyses were performed using the Duo Set Enzyme-Linked Immunosorbent Assay Kits (R&D Systems, Minneapolis, MN, USA), according to the manufacturer’s instructions. Values were estimated by interpolating data using a standard curve for each cytokine by a colorimetric assay, which was measured at 450 nm (correction at 540 nm) in a spectrophotometer (Perlong DNM-9602, Nanjing Perlove Medical Equipment Co., Nanjing, China). Values obtained were expressed in picograms per milligram (Salm et al., 2023).

### 2.9. Statistical analysis

The results were analyzed using the Graph Pad Prism software, version 8.0 (La Jolla, CA, USA). The Shapiro-Wilk test was applied for data normality, which were presented as mean ± standard error, being compared by two-way analysis of variance (ANOVA), followed by the *Post-hoc* Bonferroni’s test. In all analyses, *p*-values less than 0.05 were considered statistically significant.

## 3. Results

### 3.1. Effect of different times of pVNS associated with physical exercise on mechanical hyperalgesia

**FIGURE 2:**
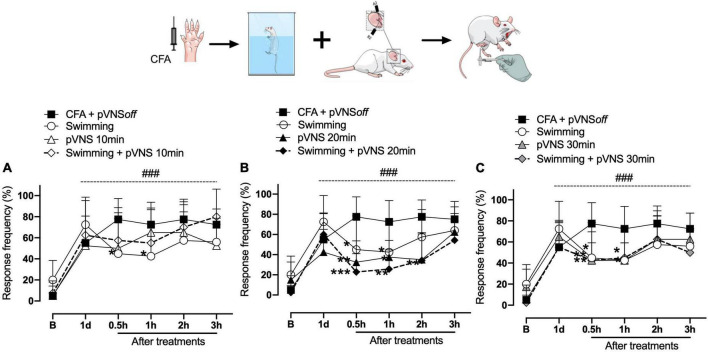
Effect of swimming associated with different times of percutaneous electrical stimulation of the auricular branch of the vagus nerve (pVNS) on mechanical hyperalgesia caused by intraplantar injection of Complete Freund Adjuvant (CFA). Groups: CFA control, swimming, pVNS 10 min and swimming + pVNS 10 min [panel **(A)**]. Groups: CFA control, swimming, pVNS 20 min and swimming + pVNS 20 min [panel **(B)**] and groups: CFA control, swimming, pVNS 30 min and swimming + pVNS 30 min [panel **(C)**] in the first day post CFA injection. Each group represents the mean of eight animals. *p < 0.05, **p < 0.01, and ***p < 0.001 when compared with the CFA control group and ^###^*p* < 0.001 when compared with the baseline **(B)**. Two-way ANOVA followed by Bonferroni’s *post-hoc* test.

**FIGURE 3:**
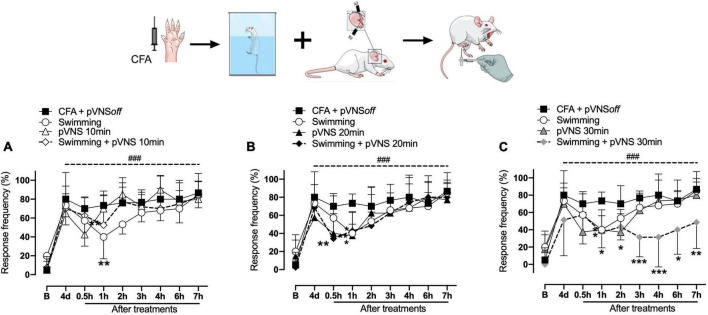
Effect of swimming associated with different times of percutaneous electrical stimulation of the auricular branch of the vagus nerve (pVNS) on mechanical hyperalgesia caused by intraplantar injection of Complete Freund Adjuvant (CFA). Groups: CFA control, swimming, pVNS 10 min and swimming + pVNS 10 min [panel **(A)**]. Groups: CFA control, swimming, pVNS 20 min and swimming + pVNS 20 min [panel **(B)**] and groups: CFA control, swimming, pVNS 30 min and swimming + pVNS 30 min [panel **(C)**] in the fourth day post CFA injection. Each group represents the mean of eight animals. *p < 0.05, **p < 0.01, and ***p < 0.001 when compared with the CFA control group and ^###^*p* < 0.001 when compared with the baseline **(B)**. Two-way ANOVA followed by Bonferroni’s *post-hoc* test.

The results shown in Figures 2, 3 demonstrate that the CFA intraplantar injection procedure induced mechanical hyperalgesia in the right hind paw of the mice. Mechanical hyperalgesia persisted throughout the period evaluated for up to 4 days after CFA injection. On the first day after CFA injection, it was observed that the swimming group, pVNS 10 min and pVNS 20 min (Figures 2A, B) presented a significant reduction in mechanical hyperalgesia at 0.5 and 1 h (*p* < 0.05) post-treatments. In the swimming group + pVNS 20 min (Figure 2B) there was a reduction in mechanical hyperalgesia at 0.5 h (*p* < 0.001), 1 and 2 h (*p* < 0.01) with greater inhibition at 0.5 h (*p* < 0.001). The pVNS 30 min and swimming + pVNS 30 min groups (Figure 2C) showed a reduction in 0.5 h (*p* < 0.01), and the swimming group + pVNS 30 min showed a reduction in mechanical hyperalgesia up to 1 h (*p* < 0.05) post-treatment.

On the fourth day, the swimming, swimming + pVNS 10 min (Figure 3A) and the pVNS 20 min (Figure 3B) groups showed a reduction in mechanical hyperalgesia in 1 h (*p* < 0.01) post-treatments. The swimming + pVNS 20 min group showed a reduction in mechanical hyperalgesia in 0.5 h (*p* < 0.01) and 1 h (*p* < 0.05). In the pVNS 30 min group, a reduction in mechanical hyperalgesia was observed at 0.5 h and 1 h (*p* < 0.05), while in the swimming group + pVNS 30 min, a reduction was observed for up to 7 h (*p* < 0.01) post-treatments (Figure 3C).

### 3.2. Effect of different times of pVNS associated with physical exercise on edema

**FIGURE 4:**
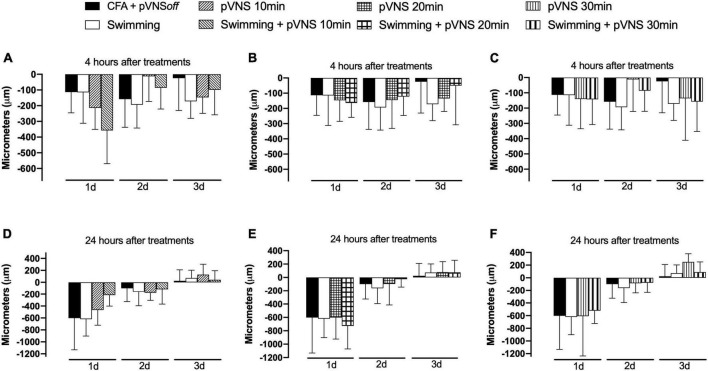
Effect of swimming associated with different times of percutaneous electrical stimulation of the auricular branch of the vagus nerve (pVNS) on paw edema by intraplantar injection of Complete Freund Adjuvant (CFA). Groups: CFA control, swimming, pVNS 10 min and swimming + pVNS 10 min [panels **(A,D)**]. Groups: CFA control, swimming, pVNS 20 min and swimming + pVNS 20 min [panels **(B,E)**] and groups: CFA control, swimming, pVNS 30 min and swimming + pVNS 30 min [panels **(C,F)**] in the first-, second- and third-day post CFA injection. Each group represents the mean of eight animals. Two-way ANOVA followed by Bonferroni’s *post-hoc* test.

The results presented in Figures 4A–F demonstrate that there was no statistically significant difference between the treatments in the reduction of edema in the paw of the animals subjected to the intraplantar injection of CFA in the right hind paw.

### 3.3. Effect of different times of pVNS associated with physical exercise on temperature

**FIGURE 5:**
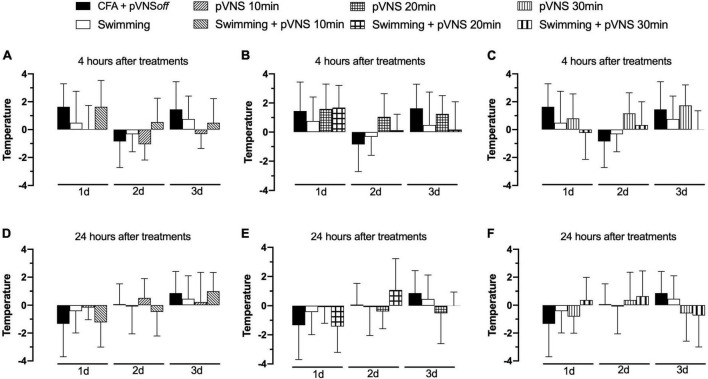
Effect of swimming associated with different times of percutaneous electrical stimulation of the auricular branch of the vagus nerve (pVNS) on paw temperature by intraplantar injection of Complete Freund Adjuvant (CFA). Groups: CFA control, swimming, pVNS 10 min and swimming + pVNS 10 min [panels **(A,D)**]. Groups: CFA control, swimming, pVNS 20 min and swimming + pVNS 20 min [panels **(B,E)**] and groups: CFA control, swimming, pVNS 30 min and swimming + pVNS 30 min [panels **(C,F)**] in the first-, second- and third-day post CFA injection. Each group represents the mean of eight animals. Two-way ANOVA followed by Bonferroni’s *post-hoc* test.

The results presented in Figures 5A–F demonstrate that there was no statistically significant difference between the treatments in the paw surface temperature of the animals submitted to the intraplantar injection of CFA in the right hind paw.

### 3.4. Effect of different times of pVNS associated with physical exercise on concentrations of inflammatory cytokines

**FIGURE 6:**
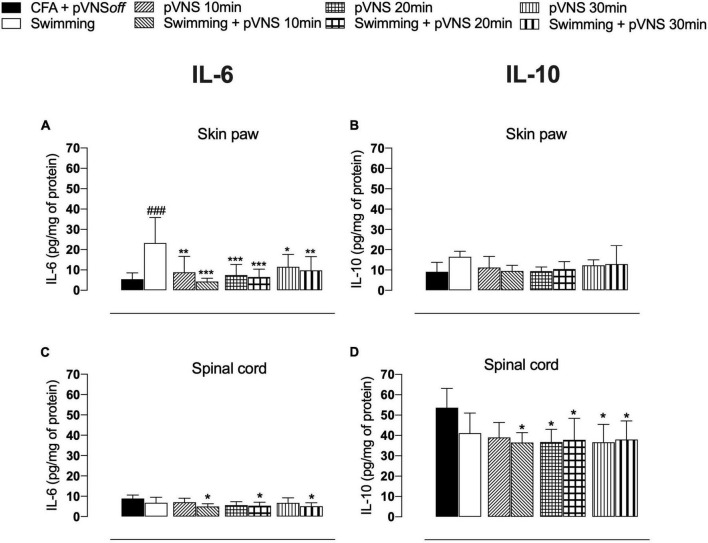
Effect of swimming associated with different times of percutaneous electrical stimulation of the auricular branch of the vagus nerve (pVNS) on cytokines levels in the paw skin [panels **(A,B)**] and spinal cord [panels **(C,D)**] interleukin-6 (IL-6) and interleukin-10 (IL-10), respectively, in mice with intraplantar injection of Complete Freund Adjuvant (CFA). Groups: CFA control, swimming, pVNS 10 min and swimming + pVNS 10 min, pVNS 20 min and swimming + pVNS 20 min, pVNS 30 min and swimming + pVNS 30 min in the fourth day post CFA injection. ^###^*p* < 0.001 when compared with the CFA control group. Each group represents the mean of eight animals. *p < 0.05, **p < 0.01, and ***p < 0.001 when compared with the swimming group. Two-way ANOVA followed by Bonferroni’s *post-hoc* test.

Four days after the administration of CFA in the paw of mice, we observed that the CFA swimming group had higher concentrations of IL-6 (*p* < 0.001) in the paw skin, when compared to the CFA control group (Figure 6A). All groups treated with pVNS at different times or associated with swimming showed lower concentrations of IL-6 (*p* < 0.01) in the paw skin, when compared to the swimming group. The treatments did not affect the concentrations of IL-10 (*p* > 0.05) in the paw skin of the mice (Figure 6B). In the spinal cord, only pVNS treatments alone decreased IL-6 concentrations when compared to the CFA control group (Figure 6C). Furthermore, the swimming + pVNS 10 min, pVNS 20 min, swimming + pVNS 20 min, pVNS 30 min and swimming + pVNS 30 min groups had lower concentrations of IL-10 (*p* < 0.05) in the spinal cord, when compared to the CFA group (Figure 6D).

## 4. Discussion

### 4.1. pVNS potentiates reduction of mechanical hyperalgesia induced by physical exercise

In the present study, we evaluated the effects of physical exercise associated with pVNS on pain and inflammation reduction in an animal model of persistent inflammatory pain. We observed that the association of therapies caused a prolonged reduction of mechanical hyperalgesia. The analgesia induced by swimming for 30 min for four consecutive days has been well documented (Kuphal et al., 2007; Mazzardo-Martins et al., 2010). The most studied hypothesis of analgesia produced by physical exercise is the release of endogenous opioids in peripheral, spinal and/or supraspinal sites, contributing to pain modulation (Thorén et al., 1990), but the participation of adenosinergic (Martins et al., 2013), serotoninergic (Mazzardo-Martins et al., 2010), noradrenergic (Mazzardo-Martins et al., 2010), and cannabinoid systems (Ludtke et al., 2020) has also been demonstrated. In this sense, Ludtke et al. (2020) demonstrated the involvement of the endocannabinoid system on the antihyperalgesic effect of physical exercise using high-intensity swimming for 30 min a day for five consecutive days. The results showed that swimming reduced mechanical hyperalgesia for up to 1 h after the end of treatment, corroborating the findings of the present study on analgesia caused by swimming (Ludtke et al., 2020).

On the other hand, physical exercise, in addition to promoting the analgesic effect, is also a potent vagal stimulator (Gourine and Ackland, 2019). Physical exercise, when practiced daily, can activate the cholinergic anti-inflammatory pathway. high levels of physical activity are associated with improved vagal tone, contributing as a therapeutic approach to resolve the inflammatory process and prevent the development of chronic diseases (Lujan and DiCarlo, 2013; Gourine and Ackland, 2019). Gourine and Ackland (2019) described in a review that possibly the strength of cardiac vagal activity directly determines the individual ability to exercise. It is already known that greater exercise capacity is strongly associated with lower resting heart rate and indirect measures of high cardiac vagal activity (Goldsmith et al., 1997). A study that used heart rate variability to measure vagal activity found that a group of untrained young people subjected to 6 weeks of aerobic training reduced heart rate at rest and increased cardiac vagal tone (Al-Ani et al., 1996). From this, it has been suggested that the vagal tonus can be modulated through the practice of physical exercise (Gourine and Ackland, 2019).

### 4.2. Swimming exercise combined with pVNS modulates inflammatory cytokines, but does not alter paw edema and temperature

The second important result reported in this study concerns the modulation of inflammatory cytokines caused by exercise combined with pVNS on the inflamed paw and spinal cord of mice. It is well established that the vagus nerve inhibits the production of pro-inflammatory cytokines, contributing to the resolution of inflammation (Rosas-Ballina and Tracey, 2009). Inflammatory cytokines are inflammatory mediators responsible for sensitizing and activating primary nociceptive neurons (Salvemini et al., 2011) that trigger pain, which is one of the most common symptoms of inflammatory disease (Ferreira, 1972). The vagus nerve can detect and control the inflammatory process via the humoral mechanism through activation of the HPA axis, which modulates the main organs of the immune system, such as the spleen and liver, sources of synthesis of pro-inflammatory cytokines (Tracey, 2009). Thus, inflammatory mediators activate afferent neural pathways of the vagus nerve, which, in turn, stimulate the release of pituitary adrenocorticotropic hormone, which prevents exacerbation of inflammation (Rosas-Ballina, Tracey, 2009). The cholinergic anti-inflammatory reflex is a neural mechanism that acts as a physiological regulator of inflammation, thus responds to environmental damage, pathogens and other external threats by activating the immune system. This mechanism is associated with acetylcholine (ACh), the main neurotransmitter of the vagus nerve. Its interaction with its α7 nicotinic receptor (α7nAChR) inhibits the synthesis of pro-inflammatory cytokines (Watkins and Maier, 1999).

In turn, ACh release is related to the splenic sympathetic anti-inflammatory pathway activated by the vagus nerve. This activation induces the release of noradrenaline by the splenic nerve, which leads to activation of the β2-adrenergic receptor in splenic lymphocytes. Activated lymphocytes then release ACh, which inhibits the release of pro-inflammatory cytokines by the macrophage (Olofsson et al., 2012). It also contributes to the change in the phenotype of macrophages M1, which have a pro-inflammatory profile, to M2, which have an anti-inflammatory action (Rasmussen et al., 2018). Recently, it was observed that VNS also contributes to the resolution of inflammation due to the production of pro-resolutive mediators, for example: resolverins, maresins, proteins, and lipoxins (Serhan et al., 2019).

It is believed that all the mechanisms mentioned above may mediate, at least in part, the results observed in the present study involving swimming associated with pVNS. Our data solely demonstrates that levels of IL-6 are increased in the of paw of mice subjected to swimming, and treatment with pVNS was able to reduce the higher IL-6 levels found in the swimming only group. Consistent with our findings, data from literature have demonstrated an increased level of this cytokine in the circulation of animals subjected to swimming-induced stress (Kapilevich et al., 2017), and also in the muscle of mice subjected to swimming exercise (Hojman et al., 2019). We also found that swimming exercise combined with pVNS reduced the concentrations of IL-10 in the spinal cord. Despite IL-10 contributing to antinociception in different models of pain in mice, the swimming exercise modulates pathways in rodents leading to a regulation of cytokines production and release (either pro- or anti-inflammatory) in the central nervous system. In fact, a decrease inIL-10 expression was found in the CNS (hippocampus) of mice subjected to swimming exercise.

In human beings, VNS through cervical stimulation for the treatment of rheumatoid arthritis reduced TNF concentrations for up to 84 days, showing improvement in the severity of the disease (Koopman et al., 2016). In addition, cervical VNS also reduced levels of pro-inflammatory cytokines in individuals with rheumatoid arthritis and Crohn’s disease, suggesting an anti-inflammatory effect of VNS (Sinniger et al., 2020; Drewes et al., 2021). Shimojo et al. (2019) demonstrated the direct relationship between physical exercise and vagal activation. They demonstrated that swimming for 1 h a day for seven consecutive days attenuated serum levels of tumor necrosis factor (TNF) in the spleen of mice compared to the control group in an endotoxemia model, an effect that was prevented by vagotomy bilateral. In humans, an inverse association was observed between the vagal tone and TNF-alpha level in Crohn’s disease patients (Pellissier et al., 2014). Physical exercise can increase vagal tone and the vagus nerve can reduce TNF production.

To date, there are few studies that have investigated the effects of physical exercise associated with VNS. In humans, the effects of physical exercise associated with pVNS for 30 min with a frequency of 10 Hz and pulse width of less than 500 ms on pain and quality of life of patients with fibromyalgia has been evaluated. The group with the combination showed improvement in quality of life (Kutlu et al., 2020). Still, another study evaluated whether the long-term use of VNS for a period of 30 min combined with an upper limb rehabilitation program is safe and effective in patients after stroke. It was observed that after 1 year there was improvement in function, in addition to demonstrating it to be a safe therapy (Dawson et al., 2020).

In this sense, VNS is already approved by the Food and Drug Administration (FDA) as a treatment for epilepsy and depression (Howland et al., 2011; Morris et al., 2013) and is currently being explored as a treatment for a variety of inflammatory diseases. Research through non-clinical studies investigate the VNS on diseases such as sepsis, lung injury, rheumatoid arthritis and diabetes, and suggest that the VNS as well as physical exercise promote an increase in vagal tone (Johnson and Wilson, 2018).

Finally, despite physical exercise associated with pVNS prolonging the reduction of mechanical hyperalgesia, there was no reduction in edema and paw surface temperature. However, of the cited studies, only Levine et al. (2014) evaluated edema in an arthritis model used, in which a significant reduction was observed after treatment with VNS. Our contradictory results with the literature may be due to the limitation of the methodology used to measure paw thickness. Thus, future histological and biochemical analyzes could help to elucidate this discrepancy.

We recognize several limitations of the present study. However, these limitations can be addressed in future studies that wish to build on this innovative proposal to enhance the benefits of exercise through auricular stimulation of the vagus nerve. The main limitation is the lack of real-time monitoring of vagal activity through recording of heart rate variability (HRV), particularly vagal tone, and the vagotomy experiment in mice. This was not performed in the present study due to its great complexity involved but future experiments can be conducted to confirm and support the role of the vagus nerve in pVNS-caused potentiation of analgesia caused by physical exercise.

In conclusion, swimming exercise combined with pVNS prolongs the anti-hyperalgesic effect of swimming, does not alter paw edema and temperature, but modulates inflammatory cytokines in the periphery and central nervous system. Finally, it is suggested that the combination of these therapies, due to their multiple benefits, can potentially be very interesting and effective, being a more integral way to manage inflammatory pain.

## Data availability statement

The raw data supporting the conclusions of this article will be made available by the authors, without undue reservation.

## Ethics statement

The animal study was approved by the Committee on Ethics in the Use of Animals (CEUA) of the University of Southern Santa Catarina (CEUA-UNISUL) under protocol 20.007.4.08.IV. The study was conducted in accordance with the local legislation and institutional requirements.

## Author contributions

AD, RL, and DM wrote the manuscript. AD, DS, BO, RS, FT, DL, EB, GB, VG, AM, FB, and JM conducted experiments. DM and WR analyzed the data. DM designed and supervised the study. All authors contributed to the article and approved the submitted version.
